# Premonitory symptoms of Feeding and Eating Disorders in pediatric age

**DOI:** 10.1186/1824-7288-41-S2-A25

**Published:** 2015-09-30

**Authors:** Giampaolo De Luca, Matteo Napoletani

**Affiliations:** 1 Pediatra di famiglia, Studio Medico Pediatrico, Amantea (CS), 87032, Italy; 2 Studente in medicina, Sapienza Università di Roma, Roma (RM), 00185, Italy

## 

Feed and Eating Disorders (FED), whose diagnostic criteria have recently been modified in the DSM-5 [1], are multifactorial diseases caused by complex interactions between biological, psychological and social factors [2,3], whose frequency is sharply increasing in adolescence [4-6].

The diagnosis is complex, especially in early adolescence (8-12 years), because of the extreme heterogeneity of symptomatic expressions, which doesn't allow a precise nosographic assignment [3,7-9]. The consequent diagnostic delay has a negative influence on the course of treatment and prognosis, making recoveries less and less frequent [10-12].

The role of the Family Pediatrics is, therefore, essential to intercept, through simple diagnostic tests (such as EAT 26) the first signs of these conditions because from this depends on the subsequent diagnosis, therapy and prognosis [13-18] (Table 1).

**Table 1:** The most significant questions in suspicion of FED among adolescent. Traits items of EAT-26 which correlate with positive total scores.

“How many diets have you begun in last year?
“Do you think, you should be on diet?”

“Do you feel dissatisfied of the weight of your body?”

“The weight influences the idea that you have of yourself?”

The first task is to suspect a FED and to assess the differential diagnosis or comorbidity with other organic or mental diseases [2,3,6,19-21] (Table 2).

**Table 2:** Differential diagnosis with other organic diseases.

Endocrine: hyperthyroidism, diabetes mellitus, Addison's disease, Simmonds syndrome
Gastrointestinal: achalasia, celiac disease, chronic inflammatory bowel disease, giardiasis and other malabsorption

Gynecological: pregnancy, other causes of amenorrhea

Infectious: AIDS, fungal infections, tuberculosis, subacute bacterial endocarditis

Neoplastic: meningiomas and any type of malignant tumor

Drugs: amphetamine, thyroid hormones, antidepressants, tricyclic, neuroleptics, lithium

The second task is to assess the severity of the problem for both organic [22] and psychic aspects, in order to formulate an operational program sustainable and shareable with the family and establish the urgency of sending the patient to the specialist and the type of taking charge (outpatient or inpatient).

We propose to distinguish three steps of increasing severity, with which FED may present themselves to the observation of the family pediatrician: the suspect, the diagnosis, the emergency.

The suspect, includes those patients who have just embarked on dangerous or insane practices to lose weight without falling in any of the diagnostic categories of DMS-5 [1]. These patients need an educational intervention that can be done by the pediatrician (Table 3).

**Table 3:** Educational Intervention by the family pediatrician (Heath budget for FED). Valuations/informations relative to the following items.

Balanced nutrition and health
Caloric needs

Satisfaction of the body image

“Necessary” and “dangerous” food

Using compensation mechanisms to bingeing (vomiting, compulsive motor activity),

Use of drugs

Family, social, emotional relations

The diagnosis, includes cases that fully meet the diagnostic criteria of DMS-5 [1], without showing signs of serious and immediate biological or psychological risk. Such patients can be initially helped through the motivational interviewing [23] and subsequently entrusted to a multidisciplinary team, which also takes care of the family, promoting inter and intra-family relationship [11,24].

The emergency, includes patients in serious condition for which is indicated urgently indicated a taking in charge by a multi-professional team, possibly with an ICU admission, inpatient or outpatient (Table 4).

**Table 4:** The indications of hospitalization.

I. Biological decompensation (includes all the serious organic conditions) a. Serious weight loss (25-40%) b. Rapidly evolutive weight loss c. Total refusal of food d. Serious complications of malnutrition as syncopations, convulsions, cardiac arrhythmias or congestive heart failure, dehydration, acrocyanosis, instability of physiological parameters (Systolic Blood Pressure ≤ 90 mmHg, Heart Rate ≤ 40 / min, body temperature ≤36 °C)
**II. Psychological decompensation** (includes all high risk situations and the psychiatric comorbidities) a. Suicide attempts b. Self-mutilations c. Abuse of drugs or other substances d. Severe depression e. Anxiety f. Obsessive-compulsive personality disorder Borderline personality disorder g. Sexual or physical abuse.

**III. Other situations** a. Failure of outpatient treatment, after attempt of 2-3 months without any modifications in the clinical picture b. Problematic family situation c. Request from the patient or from his family

Since drop-out and relapses are frequent in the course of the FED [25] remains to the pediatrician to assess the progress of the disease and the outcome of care, to manage over time any residual symptoms or relapses or even new emergencies.
